# Stress‐testing the brain to understand its breaking points

**DOI:** 10.1113/JP276184

**Published:** 2018-04-24

**Authors:** R. Ryley Parrish, Andrew J . Trevelyan

**Affiliations:** ^1^ Institute of Neuroscience Medical School, Framlington Place Newcastle upon Tyne NE2 4HH UK

**Keywords:** epilepsy, seizures, Entorhinal cortex, Inhibition

Epilepsy is a common and serious neurological condition, characterized by recurrent seizures. In fact it is one of the better managed brain disorders, with up to 70% of people acquiring good control on medication, but that still leaves huge numbers of people with uncontrolled seizures. Aside from learning how to treat these refractory cases, epilepsy retains many other mysteries: for most seizures, we have no idea what provokes them, what dictates how they spread through the brain, and why, just as suddenly, they stop, the vast majority terminating spontaneously, without medical intervention. Many, probably even most, seizures arise from a focal site, and then spread into territories that just before may have been functioning perfectly normally. Given this interplay between focal pathology and surrounding functional tissue, and the chronic nature of the condition, one might wonder what could be learnt from an acute experimental preparation that is entirely bathed in a pro‐epileptic medium. Such preparations, though, have proved to be a mainstay for epilepsy research for more than 30 years now, in particular, providing insights into the nature of paroxysmal depolarising shifts, ictogenesis and spreading ictal activity (Traub & Miles, 1991).

In this issue of *The Journal of Physiology*, Ridler *et al*. (2018) use brain slices from rodent medial entorhinal cortex (mEC) to ask how inhomogeneity in the tissue affects epileptiform discharges. In essence, by pharmacologically challenging the tissue, they were seeking to identify fault lines in the tissue, the sites where a seizure could take hold. It is akin to how an engineer might ‘stress‐test’ the parts of a ship, separate from the whole structure.

On the back of the seminal work by O'Keefe, the Mosers, and others, the entorhinal cortex has become a paradigm model for understanding cognition. In the process, many groups have noted gradients of cellular properties across this area. Its relevance to epilepsy is that entorhinal cortex is likely to be an important conduit for seizures arising in the temporal lobe, the most common site of focal epilepsy. Ridler and colleagues now show that propagation through this brain area is shaped strongly by the pattern of inhibitory connectivity; dorsal mEC stellate neurons have more inhibitory inputs compared to ventral stellate cells, and previous work has shown that this may explain a dorso‐ventral gradient in gamma oscillations (Beed *et al*. 2013). Similarly, Ridler *et al*. show that epileptiform activity arises predominantly from ventral mEC, the side with less inhibition, and spreads at a slow rate to the dorsal side.

That isn't the whole picture, though, because the same gradient in seizure susceptibility was also seen in disinhibited tissue; this was attributed to differences in intrinsic cellular properties along the dorso‐ventral axis of the mEC. The more general point is that when considering how seizures spread, we must take into account both the axonal pathways, but also how the target brain areas respond (Trevelyan, 2016); a critical feature is the endogenous protective mechanisms resisting spreading epileptiform activity that have been identified using similar *in vitro* preparations.

This study also has relevance to whether GABAergic activity promotes or restrains epileptic activity. The debate on this point has arisen in part because of model‐specific differences in how epileptiform activity develops. The low Mg^2+^ model enhances excitation, and the earliest epileptiform discharges have a very large glutamatergic component, and yet the postsynaptic response is disproportionately small, or even absent, a fact that is explained by the parallel bombardment from interneurons. In this case, what requires explanation is the minimal response in the presence of the large glutamatergic drive, and GABA is clearly acting as a restraint. In contrast, 4‐aminopyridine initially induces almost purely interneuronal bursts, which may give rise to full ictal events by chloride‐loading pyramidal neurons, with a secondary surge in extracellular [K^+^], as chloride is removed, coupled to K^+^, by the co‐transporter KCC2 (Viitanen *et al*. 2010). Interestingly, Ridler *et al*. used 4‐aminopyridine, so one might have expected events therefore to have arisen from the ‘inhibitory‐dense’ dorsal territory, but instead their data add further evidence for the restraining role of interneurons in epilepsy. Regardless of that debate, the data fully support the idea that the local microcircuitry and cellular behaviour will influence how new territories are recruited to a seizure event. There are likely to be multiple ways in which seizures start, but during the generalization process, when the seizure propagates away from the ictal focus into functional cortical areas, GABA will surely act in its traditional sense, to inhibit activity. This, though, can be short‐lived, with GABA quickly becoming excitatory (as has also been shown using the 0 Mg model; Ellender *et al*. 2014), because the combined glutamatergic and GABAergic bombardment is one of the fastest ways of chloride‐loading cells. This merely re‐emphasises the point that GABAergic inhibition is clearly a double‐edged sword.

Ridler *et al*. have provided a glimpse into the continued utility of in vitro models, to ‘stress‐test’ the brain. This approach can help us explore how different parts of the brain may vary in their susceptibility to seizure activity, arising from differences in their inputs, the local microcircuitry and cellular properties. Interpretation of the recordings, though, must focus on both commonalities and differences between the models used, especially if we are to extrapolate these findings for clinical use.

## Additional information

### Competing interests

None declared.

### Author contributions

Both authors have read and approved the final version of this manuscript and agree to be accountable for all aspects of the work in ensuring that questions related to the accuracy or integrity of any part of the work are appropriately investigated and resolved. All persons designated as authors qualify for authorship, and all those who qualify for authorship are listed.

### Funding

RRP and AJT are supported by grants from MRC (MR/R005427/1) and BBSRC (BB/P019854/1).
